# The Association of Sleep Disordered Breathing with Heart Failure and Other Cardiovascular Conditions

**DOI:** 10.1155/2013/356280

**Published:** 2013-12-17

**Authors:** Elizabeth Stopford, Karthik Ravi, Vikrant Nayar

**Affiliations:** Department of Cardiology, Pinderfields Hospital, Gate 47, Aberford Road, Wakefield WF1 4DG, UK

## Abstract

An abundance of evidence exists in support of primary and secondary prevention for tackling the scourge of cardiovascular disease. Despite our wealth of knowledge, certain deficiencies still remain. One such example is the association between sleep disordered breathing (SDB) and cardiovascular disease. A clear body of evidence exists to link these two disease entities (independent of other factors such as obesity and smoking), yet our awareness of this association and its clinical implication does not match that of other established cardiovascular risk factors. Here, we outline the available evidence linking SDB and cardiovascular disease as well as discussing the potential consequences and management in the cardiovascular disease population.

## 1. Sleep Disordered Breathing and Cardiovascular Disease

### 1.1. Opening Statement

Sleep disordered breathing (SDB), encompassing both obstructive and central sleep apnoea, has been associated with increased cardiovascular morbidity and mortality. It occurs in half of all heart failure patients and is linked to hypertension, arrhythmia, impaired glucose tolerance, cerebrovascular disease, and ischaemic heart disease [1–3]. Despite the high prevalence and significant morbidity associated with SDB, our awareness and understanding of this condition remain incomplete.

### 1.2. Introduction

An abundance of evidence exists in support of primary and secondary prevention for tackling the scourge of cardiovascular disease. Despite our wealth of knowledge, certain deficiencies still remain. One such example is the association between SDB and cardiovascular disease. A clear body of evidence exists to link these two disease entities (independent of other factors such as obesity and smoking) yet our awareness of this association and its clinical implication does not match that of other established cardiovascular risk factors [4, 5]. Here, we outline the available evidence linking SDB and cardiovascular disease as well as discussing the potential consequences and management in the cardiovascular disease population.

## 2. Sleep Disordered Breathing and Its Apnoeic Subtypes: Obstructive or Central?

Sleep apnoea can be defined as the cessation of breathing during sleep which lasts for at least 10 seconds, on five or more occasions per hour, and which leads to tiredness during the daytime [6]. Presenting symptoms may include morning headaches, unrefreshing sleep, and the effects of increased daytime somnolence (poor concentration, mood changes, and reduced libido). In over 80% of cases, however, individuals suffering from sleep apnoea will be completely unaware of their condition and it is often their partners that report classical snoring symptoms. The gold standard method for confirming nocturnal apnoea and diagnosing SDB is full polysomnography, comprising of multiple recorded variables including thoracoabdominal movement, oxygen saturation, electrocardiography and respiratory airflow. In clinical practice, however, sleep studies typically involve more time- and cost-effective methods such as pulse oximetry. A 4% or greater fall in oxygen saturations correlates with a 10-second pause or apnoea [6].

**Figure 1:**
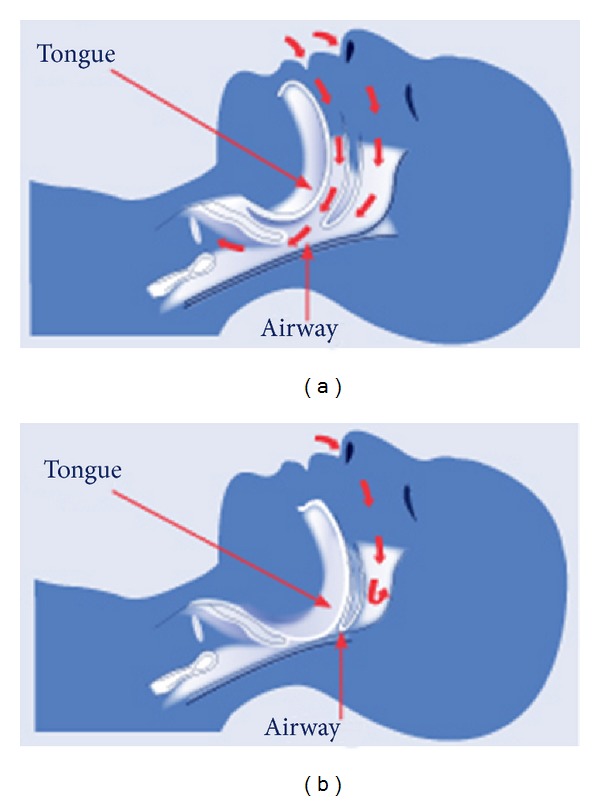
Mechanism of airway obstruction in OSA. (a) demonstrates normal breathing; (b) shows posterior movement of the tongue producing OSA.

The severity of sleep apnoea is assessed using the apnoea-hypopnoea index (AHI), which represents the number of apnoeic or hypopnoeic (reduction in oronasal airflow amplitude by ≥30% for a period of at least 10 seconds) episodes per hour of sleep. Sleep apnoea can be classified as mild (AHI = 5–15), moderate (AHI = 15–30), or severe (AHI ≥ 30) [6]. Sleep apnoea is further categorised according to the underlying mechanism of apnoea [2]. Obstructive sleep apnoea (OSA) is the most prevalent form of sleep apnoea amongst the general population and involves recurrent collapse of the pharyngeal airway leading to reduction or cessation in oropharyngeal airflow and subsequent arousal (Figure 1) [2, 4, 7]. Central sleep apnoea (CSA) is not caused by occlusion of the pharynx but rather an autonomic-mediated reduction of ventilation due to a fall in the partial pressure of carbon dioxide below the threshold needed to stimulate breathing. Once this hypocapnic threshold is breached, ventilation decreases and the carbon dioxide level increases. This homeostatic mechanism maintains stable levels of serum carbon dioxide and pH [2].

A combination of the two apnoeic pathologies can occur in some patients, defined as mixed sleep apnoea. Obstructive apnoeas can lead to an impaired homeostatic response to hypercapnia and the subsequent development of central apnoeas. This happens primarily in heart failure patients with the exact mechanism not fully understood. Obstructive and central apnoea can also combine to produce complex sleep apnoea. This occurs when patients with OSA treated with continuous positive airway pressure (CPAP) convert to central apnoeic episodes; again believed to be a consequence of impaired homeostatic mechanisms [8].

## 3. How Is Sleep Disordered Breathing Related to Cardiovascular Disease?

The prevalence of SDB in the general population is estimated to be between 5 and 10% [4, 6, 9]. This figure rises to be between 30–83%, 30–58%, and 50–80% in hypertensive patients, ischaemic heart disease patients, and the heart failure population, respectively [5, 10, 11]. Evidence supports the role of SDB in the development and progression of cardiovascular disease and vice versa. The exact mechanisms for this association are not fully defined but have been widely postulated.

## 4. Pathophysiology: How Does SDB Contribute to Cardiovascular Disease?

**Figure 2:**
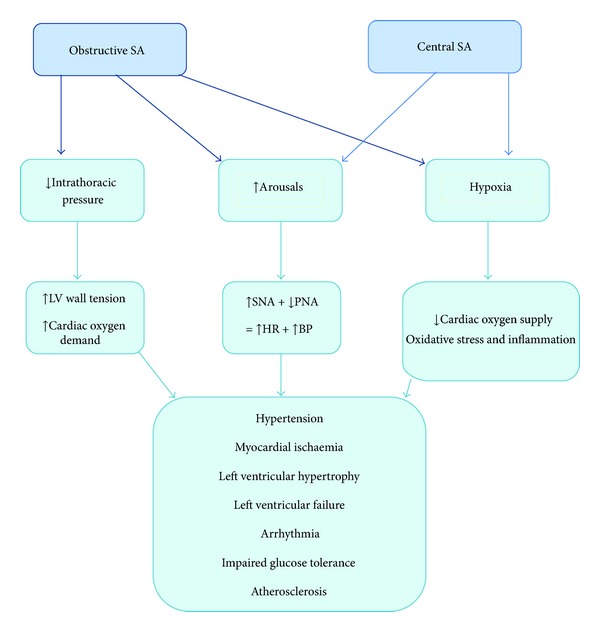
Summary of the physiological mechanisms contributing to cardiovascular disease in SDB. SA: sleep apnoea; SNA: sympathetic nervous activity; PNA: parasympathetic nervous activity.

Normal sleep physiology involves a reduction in sympathetic nervous system activity combined with an increase in parasympathetic tone. The combined effect is a decrease in heart rate, stroke volume, and systemic vascular resistance so reducing cardiac output, blood pressure, and myocardial workload during sleep. In SDB, this usual cardiovascular quiescence is disrupted through the mechanisms outlined below (Figure 2) [5].

*(1) Hypoxia*. Hypoxic episodes may directly impair cardiac contraction and diastolic relaxation by reducing oxygenation and hence cardiac efficiency. In addition, intermittent hypoxia may create oxygen-free radicals and activate inflammatory pathways so causing hypertension by direct effects on vascular endothelium, independent of sympathetic tone [11].

*(2) Repeated Arousals during Sleep.* In SDB, termination of apnoeic episodes is accompanied by a sudden and brief arousal from sleep, with an accompanying increase in sympathetic activity, reduction in vagal tone, and resultant peripheral vasoconstriction [5, 12]. This is evidenced by an increase in urinary catecholamines in patients with untreated OSA, which returns to normal levels after effective therapy [3]. Therefore, during sleep patients will experience surges in heart rate and blood pressure accompanying brief episodes of arousal.

*(3) Exaggerated Negative Intrathoracic Pressure.* In OSA there is ineffective inspiration due to attempts to inspire against a fully or partially collapsed pharynx, so increasing the inspiratory negative intrathoracic pressure. The resultant greater venous return to the right side of the heart increases cardiac preload and work [5].

## 5. Sleep Disordered Breathing: The Clinical Implications

The causative mechanisms of obstructive and central type sleep apnoea differ, but the physiological and hence cardiovascular sequelae can be considered in unison [12]. However, there are several difficulties with definitively relating the physiological changes associated with SDB to the development and progression of clinical cardiovascular disease. Firstly, a multitude of confounding factors are shared between the two. For example, obesity is a common characteristic of patients suffering from sleep apnoea (specifically OSA) but is also implicated in the development of cardiovascular conditions such as hypertension and ischaemic heart disease [3, 4]. Complicatedly, CSA arises as a *consequence* of heart failure, and once present, may further exacerbate cardiovascular disease progression [2, 4]. Secondly, studies have suffered from small sample population sizes and differing inclusion criteria and methodology [1, 4, 13]. However, despite these difficulties, there is increasing evidence supporting the causal relationship between SDB and the subsequent development and progression of cardiovascular disease that is independent of other factors [1, 4, 11].

## 6. Hypertension

The repeated arousals that occur with apnoeic episodes result in an increase in heart rate and blood pressure, typically 5–7 seconds following an apnoea [9, 14]. Evidence indicates that the repeated apnoeas not only cause the temporary nocturnal increases in blood pressure but, over time, inflict chronic alterations in blood pressure leading to persistent daytime hypertension. Indeed, incident hypertension has been found to be three times as common in SDB patients as compared with that in the general population [14]. Alongside increased sympathetic tone, apnoea increases the expression of vascular adhesion molecules, alters cytokine levels, promotes oxidation of low density lipoproteins, and impairs endothelial cell function, which may further augment hypertension and contribute towards an atherosclerotic process [5].

A cross-sectional analysis of 6,132 subjects who underwent polysomnography at home showed a definite association between OSA and hypertension with the prevalence of hypertension increasing in proportion to the AHI [15]. A further prospective study revealed a 2.89-fold increase in hypertension for AHI = 15 compared with AHI = 0 over a 4–8-year time period [14]. Studies employing animal models have also demonstrated this relationship. Dogs exposed to repeated obstructive episodes over a 1–3-month period developed a significant increase in blood pressure during sleep and wakefulness [16].

OSA may also predispose to resistant hypertension defined as a blood pressure persisting above 140/90 mmHg despite lifestyle intervention and the use of three or more pharmacological agents, one of which should be a diuretic [17]. Greater than 60% of patients with resistant hypertension are at high risk for OSA in accordance with the Berlin Questionnaire, but the actual polysomnographic diagnosis is greater; about 70% of females and 90% of males with resistant hypertension have OSA [18–20]. Combinations of mechanisms mediated through OSA are likely to be responsible for the predisposition to resistant hypertension. These may include increased levels of endothelin (a potent vasoconstrictor), hyperaldosteronism, and increased sympathetic drive. Notably, patients with resistant hypertension with a high risk of OSA are almost two times more likely to have primary hyperaldosteronism [21].

There is accumulating evidence in support of CPAP in the management of resistant hypertension. The use of nightly CPAP for a two-month period can produce significant reductions in both systolic and diastolic BP in patients with resistant hypertension and, similarly, compliance with CPAP may normalise nocturnal blood pressure patterns [21, 22]. However, these studies are relatively small, with most of the current evidence for CPAP reserved for the general hypertensive population rather than treatment-resistant hypertension.

## 7. Pulmonary Hypertension

As well as systemic hypertension, pulmonary hypertension is associated with sleep disordered breathing. This is likely to be via similar mechanisms as described for systemic hypertension including the effects of increased sympathetic activity. Additionally, negative intrathoracic pressure during apnoeic or hypopnoeic episodes amplifies venous return to the right heart accentuating pulmonary artery blood flow. Alongside preload, increased afterload due to pulmonary arteriolar remodelling and increased reactivity to hypoxia is also thought to contribute [23].

Pulmonary hypertension, which tends to be mild, may have a prevalence of up to 40% in the OSA population with evidence to support using nocturnal CPAP for reducing pulmonary artery pressures [23]. A study of 49 patients with polysomnography proven OSA but with normal lung function tests found that 6 patients had evidence of pulmonary hypertension at rest (defined as mean pulmonary artery pressure >20 mmHg) whilst 39 patients developed pulmonary hypertension during exercise. 25 of these 39 patients had elevated pulmonary capillary wedge pressures whilst none had raised pulmonary vascular resistance. Therefore, mild pulmonary hypertension commonly occurs in OSA patients, especially during exercise, and is probably mediated by left ventricular (LV) diastolic impairment resulting in raised pulmonary capillary wedge pressures [23, 24]. Conversely, a quarter of patients with pulmonary hypertension may suffer from OSA. A recent study assessed 169 patients with known pulmonary hypertension for SDB. 45 patients (27 OSA, 18 CSA) were found to have AHI > 10, with a mean of 20 [25].

## 8. Ischaemic Heart Disease

The link between SDB and coronary artery disease (CAD) is primarily derived from studies of OSA. Following correction for confounding factors such as age, sex, BMI, hypertension, hypercholesterolaemia, diabetes mellitus, and current smoking, the prevalence of CAD is still higher in patients with OSA. The prospective Sleep Heart Health Study revealed that subjects with a moderate to high AHI (>11) were 1.42 times more likely to develop CAD when compared with patients in the lowest AHI quartile. Likewise, there is a significantly higher prevalence of OSA in patients with angiographic evidence of CAD when compared to control patients with normal coronary arteries. The overall prevalence of OSA in the CAD population is estimated to be in the region of 30 to 58% compared with 2–4% for the general population [26–29].

Patients with SDB are more likely to have cardiac events during nocturnal hours [30]. Acute coronary syndromes (ACS) are more frequent during night time in OSA patients as opposed to the well-documented diurnal pattern of presentation in the general population [31]. The converse relationship is also true. In a study of 19 patients with sleep-onset ACS, 89% were found to have moderate or severe SDB (AHI > 15) [32]. Although an abundance of evidence exists to support the relationship between OSA and ischaemic heart disease, it is less clear whether this association is due to a direct effect of the apnoeic episodes on the myocardium during sleep or whether it is a consequence of the combined pathological processes induced by repeated apnoeas over time. A community-based study of patients without CAD has demonstrated elevated background levels of high-sensitivity troponin in 65% of subjects with OSA. Troponin levels correlated directly with severity of OSA and prognosis [33]. An earlier study of OSA patients discovered that ST-depression during sleep was relatively common and that a significant reduction in ST-depression could occur on application of nasal CPAP [34].

The effect of hypoxia on the cardiac function is not as straightforward as it may first appear. Significant research data advocates exposing the heart to repeated episodes of hypoxia to protect it from ischaemic damage through preconditioning of the myocardium. This process aids to regulate critical transcription factors within the cardiac endothelium and also promotes development of coronary collaterals, a phenomenon well-documented in patients with chronic myocardial ischaemia [35, 36].

The development of hypertension, of which SDB may be a causative factor, along with LV hypertrophy which is also associated with SDB may further accentuate the relationship between CAD and SDB; both conditions act as risk factors for ischaemic events. In addition, hypoxia induces the release of inflammatory mediators such as C-reactive protein, fibrinogen, interleukin-6, and adhesion molecules, all of which have a postulated role in the development of CAD [3, 5]. Coronary thrombus may also arise secondary to a hypercoagulable state induced via a number of mechanisms:

1. superoxide release from neutrophils depleting nitric oxide bioavailability,
2. increased catecholamine levels promoting platelet activation and aggregation,
3. hypertension induced hypercoagulability.

## 9. Heart Failure

Current evidence strongly supports an association between heart failure and SDB. Studies consistently report SDB prevalence of 50% or higher in the chronic heart failure population [5, 12, 37]. Both types of apnoea syndrome are implicated in heart failure. In one study, very severe SDB (AHI > 44 per hour) occurred in 49% of patients (37% CSA, 12% OSA) with a LV ejection fraction of less than 45% [38]. The particularly high prevalence of SDB in this population can be attributed mainly to CSA which arises as a *consequence* of heart failure [4]. CSA develops in heart failure patients through increased LV filling pressures that lead to pulmonary congestion and activation of irritant receptors within the lungs, stimulating hyperventilation and producing hypocapnoea [12]. Once carbon dioxide levels fall below a critical threshold, breathing ceases. A self-perpetuating cycle of hyperventilation-apnoea episodes ensues, typically associated with frequent arousals from sleep [2].

Heart failure, in turn, may worsen as a consequence of SDB. Right ventricular (RV) preload is increased (as discussed earlier) and hypoxia induced pulmonary arterial vasoconstriction produces increased RV afterload; the combined effect results in RV distension, a leftward shift of the interventricular septum, and a subsequent impedance to LV filling and diminution of stroke volume. Similarly, increased systemic vascular resistance may impair LV function [5].

Outcomes are worse in heart failure patients with SDB. The 3-year mortality rate in heart failure patients with untreated SDB is double that of non-SDB subjects, even when allowing for age, LV ejection fraction, and New York Heart Association functional class [39]. Similarly, the median survival of systolic heart failure patients with CSA is 45 months compared with 90 months for those without CSA. Even for patients with mild heart failure the concurrent incidence of SDB significantly increases mortality [40]. Patients with mild heart failure and CSA have a 39% mortality rate at five years compared to 11% in non-SDB patients with mild heart failure [41]. The aetiology of heart failure may also determine the risk from underlying SDB. In a prospective analysis, the presence of moderate to severe sleep apnoea (AHI ≥ 15) in ischaemic heart failure significantly increased mortality when compared to those without SDB, whilst no appreciable difference was demonstrated in nonischaemic cardiomyopathy patients [42].

The effect of cardiac resynchronisation therapy (CRT) in patients with severe heart failure and SDB has also been studied. CRT decreases the number of apnoea-hypopnoea episodes, reduces the maximum apnoea-hypopnoea duration, and results in higher minimal oxygen saturations. These physiological benefits are not seen in the OSA or patients without SDB. The benefit seen in CSA appears to be dependent on a good clinical and haemodynamic response to CRT [43, 44].

## 10. Arrhythmias

Apnoeic episodes have a tendency to induce bradycardias through increased vagal activity, whilst the subsequent postapnoea period can be associated with tachycardic episodes due to hyperventilation and sympathetic stimulation [12, 13]. The repeated surges in sympathetic nervous activity have been shown to precipitate abnormal remodelling of the atrium which in turn predisposes to the development of supraventricular arrhythmias [14]. Indeed, atrial fibrillation (AF) is four times more prevalent in patients with severe OSA (AHI > 30) than in matched patients without SDB [29]. In a recent study, twelve-month recurrence rates of AF following successful cardioversion were 82% for patients with untreated SDB, 42% for OSA patients treated with CPAP, and 53% in the control group that never had a sleep study, thereby advocating CPAP for the prevention of recurrent AF [45]. Separately, atrial overdrive pacing was previously attempted to ameliorate CSA by preventing bradycardia and augmenting cardiac output, but this effect was not reproducible [46, 47].

The risk of ventricular arrhythmias increases with SDB. Polysomnograms have shown an almost 18-fold increase in the relative risk of arrhythmias within 90 seconds of a respiratory disturbance compared with normal breathing, with nonsustained ventricular tachycardia making up 76% of the arrhythmic episodes [48]. Also, SDB significantly increases the risk of ventricular arrhythmias in patients with implantable cardioverter-defibrillators (ICD). A prospective study of appropriate ICD therapy (antitachycardia pacing or shock therapy for ventricular tachycardia or ventricular fibrillation) over a one-year period in patients with SDB (*n* = 26) and without SDB (*n* = 19) demonstrated significantly more appropriate ICD therapies for patients with SDB (73% versus 47%). This was solely due to an increase in arrhythmias occurring between midnight and 6 a.m. with no difference in ICD therapies during waking hours [49].

## 11. Endothelial Dysfunction

Chronic intermittent hypoxia can contribute to endothelial dysfunction independent of other risk factors [50–52]. Endothelial dysfunction encompasses prothrombotic and inflammatory processes, impairment of endothelial repair mechanisms related to reduced nitric oxide availability, and increased vasoconstriction. Increased serum levels of adhesion molecules and inflammatory markers including ICAM-1, VCAM-1, E-selectin, and CRP have been measured in subjects with moderate-severe OSA independent of coexisting cardiovascular risk factors such as diabetes mellitus, hypertension, and smoking status [50]. Intermittent hypoxia caused by OSA promotes oxygen radical formation that leads to activation of transcription factors that upregulate the expression of adhesion molecules [52]. The role of these adhesion molecules is well established in the process of atherosclerosis. Also, there is reduced expression of proteins that regulate production of endothelial nitric oxide (eNOS) as well as the increase in the markers of inflammation and oxidative stress (cyclooxygenase-2, inducible NOS, and nitrotyrosine) in subjects with OSA compared to control subjects [50, 53, 54]. Adherence to CPAP for 4 weeks can reverse the downregulation of eNOS and the upregulation of markers of oxidative stress and inflammation with normalisation of circulating and exhaled markers of oxidative stress in patients with OSA [52, 55].

## 12. Cerebrovascular Disease

OSA is an independent risk factor for stroke. Patients presenting following a stroke or transient ischaemic attack are 3 to 4 times more likely to have OSA than matched control subjects, and silent infarction (infarction in the absence of stroke symptoms) occurs in 25% of OSA subjects compared with only 6.7% of control subjects [56, 57].

The brain requires a steady and continuous supply of oxygen in order to function. Measurements of cerebral blood flow velocity (middle cerebral artery) in subjects with OSA during apnoeic episodes demonstrate an increase in flow during the apnoea itself with a resultant decrease below baseline velocity on termination of the apnoea, which typically persists for one minute before return to baseline. During these hypoxic episodes, levels of brain adenosine triphosphate have been shown to decrease whereas inorganic phosphate levels increase [58]. These transient changes in cerebral blood flow may have detrimental effects on memory, spatial learning, and attention, particularly in patients with moderate-severe OSA [59–61]. In addition to the *transient* alterations in cerebral blood flow velocity during apnoeic episodes, changes in autoregulation may produce a more *permanent* impairment of cerebral blood flow velocity. This is associated with a higher incidence of stroke as well as poorer outcomes after stroke. An impaired cerebral blood flow response to changes in arterial pressure, particularly the sharp decrease in blood pressure following apnoea, can leave the brain vulnerable to ischaemia [62, 63].

In addition to changes in cerebral blood flow velocity and autoregulation, the cerebral vasculature will also be exposed to the same cascade of events that have been discussed regarding the peripheral vasculature: prothrombotic and inflammatory changes, vasoconstriction, and endothelial dysfunction. Therefore, the cerebral vascular system is also predisposed to atherosclerotic processes in the same way as the peripheral system. Specific to the obstructive form of sleep apnoea, there is evidence to suggest that physical vibration of the carotid arteries during snoring can also contribute to endothelial damage and atherosclerosis [64].

## 13. Does Treating SDB Improve CVD Outcomes?

Current evidence is largely based on observational studies with a dearth of robust randomised control trial data.

CPAP is a recognised treatment for the symptoms of OSA and has a clear role in the management of acute heart failure. Fixed pressure CPAP has consistently resulted in improved left ventricular ejection fraction (LVEF) and a decrease in sympathetic nervous system activation in patients with OSA [65]. A one-month randomised controlled trial of CPAP in patients with heart failure and severe OSA showed an increase in LVEF of 9% in the CPAP arm [66]. This was reaffirmed by a significant 2.5% increase in LVEF in heart failure patients (baseline LVEF < 45% and AHI > 10) after three months of optimal CPAP therapy. The benefit from CPAP therapy was significantly greater in individuals with a baseline LVEF >30% in this study. However, Epworth Sleepiness Scale Scores (a score from 0 to 24 which attempts to measure subjective daytime sleepiness based on patient responses to an eight-stem questionnaire), quality of life, New York Heart Association functional class, and 6-minute walking distance did not show significant improvement [67].

CPAP also appears to improve patient outcomes. Long-term (>5 years) prospective observational studies of middle-aged and elderly patients with OSA suggest increased mortality and risk of cardiovascular events in patients untreated with CPAP compared to those on treatment. The risk of cardiovascular events is not increased in patients with OSA treated with CPAP when compared to patients without OSA, so implicating a cardioprotective role for CPAP [5, 68, 69]. The short-term improvements in blood pressure, pulmonary hypertension, burden of AF, and coronary ischaemia have already been discussed but longer-term data is awaited.

CSA has been identified as a poor prognosticator in heart failure. There is less data to support CPAP in the context of CSA but application of CPAP can reduce the frequency of central apnoeic episodes and improve quality of life. A randomised controlled trial of CPAP for 66 heart failure patients (29 with and 37 without CSA) over a five-year period demonstrated a significantly reduced mortality and cardiac transplantation rate for patients with CSA receiving CPAP. CPAP did not affect mortality or the cardiac transplantation rate in subjects without CSA [70]. These findings were not subsequently replicated in a larger randomised controlled trial in which 258 heart failure patients with CSA were randomly assigned to a CPAP arm or a non-CPAP arm. Although follow-up demonstrated significant improvement in exercise capacity, apnoeic episodes, oxygen saturations, norepinephrine levels, and LVEF, the overall morbidity (cardiac transplant rate) and mortality were not significantly different between the two groups. Post hoc analysis revealed a possible improvement in transplant-free survival if the AHI was suppressed below 15. Therefore, the data remains equivocal in support of CPAP improving life expectancy in heart failure patients with CSA [71, 72].

Despite the lack of evidence for using positive airway pressure treatment to improve long-term outcomes in heart failure patients with CSA, the reduction in apnoeic episodes and improvement in symptoms merit continued CPAP usage. A 2012 meta-analysis of the available literature for the treatment of CSA recommends the use of CPAP as well as positive airway pressure delivery using an adaptive servoventilator (ASV) [73]. ASV is a device that continuously monitors the patient's breathing pattern enabling delivery of ventilatory support on detection of reduced or absent breathing and withdrawal of this support once the patient's breathing has normalised. There is efficacy of ASV in heart failure patients with CSA and, in particular, improvement in patient compliance compared with CPAP, which is often poorly tolerated. Indeed, there is alleviation of SDB in heart failure patients with OSA treated with CPAP or CSA treated with ASV as well as improvement in LV systolic function, RV systolic function, and reverse LV remodelling consistent with overall enhancement of cardiac performance [74]. Although there is evidence to support the use of CPAP and ASV in patients with heart failure, the studies to date are relatively small and of short duration. For these findings to be transferrable to wider clinical practice more robust and larger scale studies are required to address the longer-term effects on morbidity and mortality [75].

## 14. Conclusions

The prevalence of SDB is increased throughout the cardiovascular disease spectrum when compared to the general population and its presence is associated with poor prognostic outcomes. Despite the growing body of evidence illustrating this point, SDB is still underdiagnosed; research indicates that over 85% of individuals with clinically significant and treatable SDB have never been diagnosed and its relationship with cardiovascular disease states is poorly recognised [4, 5, 12]. In raising awareness of this relationship, a new target in the prevention and management of cardiovascular disease is borne. The management of SDB with positive pressure ventilation can improve not only the symptoms of sleep apnoea but also cardiovascular disease outcomes, especially for patients with heart failure. However, there is a need for larger studies (especially for CSA and mixed sleep apnoea) to confirm the long-term effects of positive pressure ventilation on cardiovascular morbidity.
